# Pollutant accumulation and microbial community evolution in rain gardens with different drainage types at field scale

**DOI:** 10.1038/s41598-023-48255-6

**Published:** 2024-01-16

**Authors:** Zhaoxin Zhang, Yang Zhang, Jiake Li, Yingying Sun, Zhe Liu

**Affiliations:** 1 Institute of Land Engineering and Technology, Shaanxi Provincial Land Engineering Construction Group Co., Ltd., Xi’an, 710075 China; 2 Key Laboratory of Degraded and Unused Land Consolidation Engineering, Ministry of Natural Resources, Xi’an, 710075 China; 3 Shaanxi Key Laboratory of Land Consolidation, Chang’ an University, Xi’an, 710064 China; 4 State Key Laboratory of Eco-Hydraulics in Northwest Arid Region of China, Xi’an University of Technology, Xi’an, 710048 China

**Keywords:** Microbial ecology, Environmental impact

## Abstract

Rain gardens play a key role in urban non-point source pollution control. The drainage type affects the infiltration processes of runoff pollutants. The soil properties and microbial community structures were studied to reveal the stability of the ecosystem in rain gardens with different drainage types under long-term operation. The results showed that the soil water content and total organic carbon in the drained rain gardens were always higher than that of the infiltrated ones. With the increase in running time, the contents of heavy metals in rain gardens showed significant accumulation phenomena, especially the contents of Zn and Pb in drained rain gardens were higher than that in infiltrated ones. The accumulation of pollutants resulted in lower microbial diversity in drained rain gardens than in infiltrated rain gardens, but the microbial community structures were the same in all rain gardens. The effects of drainage type on microbial community evolution were not significant, only the accumulation of heavy metals led to changes in the abundance of dominant microorganisms. There were differences in the soil environment of rain gardens with different drainage types. The long-term operation of rain gardens led to fluctuations in the soil ecosystem, while the internal micro-ecosystems of the drained rain gardens were in unstable states.

## Introduction

Urban stormwater management has become the key issue that must be faced with the rapid development of urbanization. Due to the rapid expansion of the impervious area of the city, the infiltration of stormwater is not possible and the infiltration amounts are reduced^1^. These phenomena will lead to an increase in urban rainfall runoff and drainage load, which will lead to problems such as urban waterlogging and urban non-point source pollution^2^. Low impact development (LID) technology has been widely used in urban stormwater management, and can effectively solve the problems such as urban waterlogging^3^. LID technology can purify and recycle stormwater runoff as a resource. LID technology carries out land planning and site design by integrating with nature, and constructs stormwater treatment facilities with drainage function (high-efficiency infiltration performance) and natural landscape features^4^. Rain gardens, which can directly be built in any unpaved space and planted with native plants, have strong runoff treatment capabilities, groundwater recharge capabilities, and landscaping capabilities^5^. Because rain gardens are simple in operation and management, and have good ecological benefits and landscape effects, they have attracted increasing industry attention^6^.

The construction of rain gardens is based on the comprehensive effects of soil, plants, and microorganisms. Therefore, many current studies have been carried out on factors that affect the treatment effect of rain gardens, such as media, plants, and structures^7^. The media in rain gardens mainly include natural materials, industrial by-products, and man-made materials. The selection of media in rain gardens mainly considers its hydraulic conductivity and pollutant adsorption capacity^8,9^. By carrying out experiments of different scales, the hydraulic parameters of different media and their ability to purify pollutants are revealed, to establish an efficient rain garden media system^10^. Jiang et al.^11^ studied the hydraulic parameters of 10 different media and their ability to remove nitrogen and phosphorus. Considering the infiltration performance, adsorption saturation capacity, and cost, fly ash, green zeolite, and water treatment residuals showed better application effects and were worth promoting. The choice of plants in rain gardens is also very important, due to the differences in the ability of plants fix carbon and nitrogen, and the effect of different plants on soil permeability and water conductivity^12^. Zuo et al.^13^ studied the nitrogen removal effects of three types of plants (*Iris pseudacorus*, *Canna indica*, and *Lythrum salicaria*), and the results showed that the removal effects of ammonia nitrogen, nitrate nitrogen, and total nitrogen by different plants were quite different. For the studies of rain garden structure, it mainly includes the depth of the media layer^14^, the setting of the internal water storage area^15^, the depth of the depression, and the type of drainage system^16^.

Groundwater recharge is one of the construction goals of rain gardens, so whether the rain gardens build a drainage system will affect its effect on groundwater recharge. In the construction of rain gardens, according to the different drainage types, they can be roughly divided into two categories: drained (impermeable) rain gardens and infiltrated (undrained) rain gardens. The infiltrated rain gardens need to fully consider the infiltration capacity of the media, while the drained rain gardens fully consider the water output capacity of the media itself. Infiltrated rain gardens have no drainage system, and the inflowing rainfall runoff seep into the surrounding soil, replenishing the over-exploited groundwater resources^17^. However, the experimental-scale studies involved so far assume that rain gardens have drainage systems^18^. Drained rain gardens can recycle rainfall runoff or discharge it evenly as surface water. Differences in drainage types can lead to differences in stormwater retention capacity and pollutant removal in rain gardens^19^, but studies on the differences between drained and infiltrated rain gardens are rare.

Rain gardens mainly remove runoff pollutants through internal matrix adsorption/retention, soil microbial degradation, and plant absorption. Referring to the existing operation and maintenance experience, rain gardens can rely on adsorption and filtration to reduce the runoff pollutants. Due to their weak internal natural degradation, rain gardens cannot achieve complete purification of pollutants^20^. Rain gardens that are arranged around roads and receive runoff with a high degree of pollution often accumulate nutrients, heavy metals, and organic pollutants^21,22^. The accumulation of pollutants in the rain gardens makes the original purification function turn into a potential source of pollution, causing lasting ecological risks to the surrounding environment^23^. For the internal habitat of the rain gardens, the soil/media of the rain gardens presents a random dry–wet alternating process due to climate change and rainfall characteristics all year round^24^. The process plays a particularly important role in the purification of the adsorbed/retained pollutants in the rain gardens, that is, the pollutants are adsorbed/retained during rainfall periods, and degraded by the functional microorganisms and plants inside the rain gardens during non-rainfall periods^25^. However, this high frequency of soil moisture fluctuations may cause irregular and extreme water stress to soil microorganisms. Simultaneously, the accumulation of pollutants from stormwater runoff can alter the original habitat in the soil and affect indigenous microbial communities^26^. Hong et al.^27^ studied the changes in the microbial community structure of rain gardens, and the results showed that the characteristics of influent water quality, biological stability of facilities, and vegetation types were important factors affecting microbial growth in rain gardens. Current studies have ignored the effects of the long-term operation of rain gardens on pollutant accumulation and internal habitats and did not address the relationship between pollutant accumulation and microbial community evolution in rain gardens with different drainage types.

In general, microorganisms in the soil/media of bioretention facilities play a larger role in the removal of pollutants, including microbial metabolism and the combination with plants growing in the rain garden. Since rain gardens with different drainage systems are bound to cause different moisture content and accumulation levels of pollutants in the soil, this also affects the functional microorganisms in the soil. Therefore, this paper aimed at rain gardens with different drainage types in Northwest China as the research objects, through long-term monitoring, mainly analyzed: (1 the temporal changes of typical pollutant accumulation levels in the soil of rain gardens with different drainage types under long-term operation; (2) the evolution of soil microbial communities; (3) the quantitative relationship between soil properties and microbial communities.

## Materials and methods

### Site description

**Figure 1:**
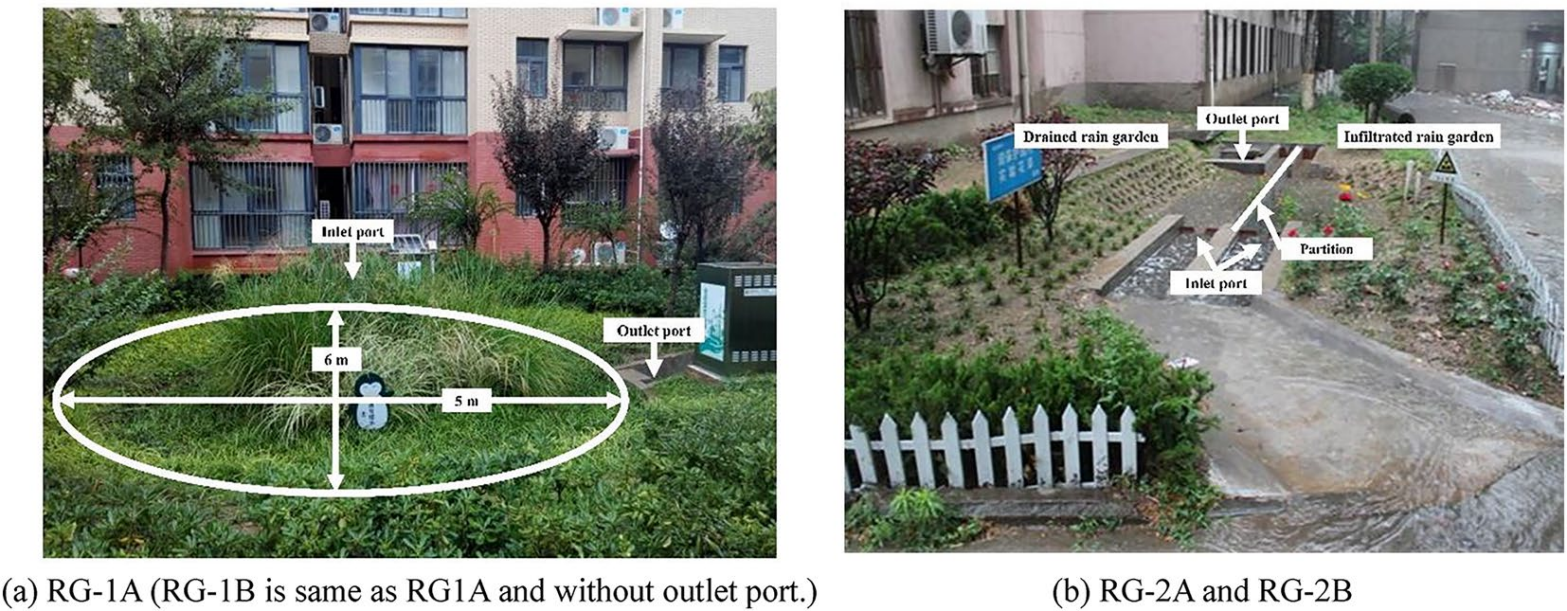
The site photos of the rain gardens.

The rain gardens involved in this study are all located in the Xi’an region in northwest China, which belongs to warm temperate semi-humid monsoon climate. The four seasons of Xi’an are warm and dry, with an average annual temperature of 13.6 °C and an average precipitation of 520 mm, and the flood season is from June to September. RG-1A/RG-1B is located on a community in Xixian New Area, and RG-2A/RG-2B is located on a university campus in Xi'an urban area. RG-1A/RG-1B were built in 2014, and is of the same scale as two oval gardens with a long axis of 6 m and a short axis of 5 m. RG-1A is a drained rain garden, with its bottom treated with a waterproof geomembrane, and the perforated drainage pipe is installed. The infiltrated stormwater in RG-1A enters the outflow triangular weir through the bottom perforated drainage pipe. RG-1B is an infiltrated rain garden, while stormwater directly infiltrates to recharge groundwater. RG-2 was built in 2012, with an overall length of 6 m, a width of 4 m, and a depth of 1.1 m. The garden is divided into two rain gardens (RG-2A/RG-2B) with the same area by a partition. RG-2A is a drained rain garden, and RG-1B is an infiltrated rain garden. The properties of the facilities were summarized in Table 1. Figure 1 shows the site photos of the rain gardens.

**Table 1:** Design parameters and size of rain gardens.

Rain gardens	Construction time	Stormwater source	Garden area (m^2^)	Catchment area (m^2^)	Discharge ratio	Media	Infiltration rate (m/s)
RG-1A/RG-1B	November, 2014	Roof	23.55	282	12:1	Planting soil	5.7 × 10^–6^
RG-2A/RG-2B	October, 2012	Road & Roof	16	155.84	15:1	Planting soil	1.8 × 10^–6^

### Rainfall events monitoring and water sample collection

The monitoring of rainfall events begins after each rain garden was constructed, and until October 2020. Among them, RG-1A/RG-1B were monitored from May 2015 to October 2020, and RG-2A/RG-2B were monitored from May 2013 to October 2020. The rainfall monitoring points are the inflow, outflow, and overflow of rain gardens, with the records of the instantaneous flow of the inflow, outflow, and overflow under each rainfall time. Water sample collection was carried out by manual sampling, and water samples were collected in the first two hours of the rainfall event (0, 5, 10, 20, 30, 60, 90, and 120 min, respectively), approximately 500 mL per water sample. The measurement indicators of water samples include suspended solids (SS), total nitrogen (TN), total phosphorus (TP), ammonia nitrogen (NH_3_–N), nitrate nitrogen (NO_3_–N), chemical oxygen demand (COD), and heavy metals including copper (Cu), zinc (Zn), chromium (Cr), and lead (Pb). All the water quality indicators were determined according to standard methods for the determination of water and wastewater^28^. The content of TN was determined by the alkaline potassium persulfate digestion method, NH_3_–N was determined by the phenol disulfonic acid spectrophotometry method, NO_3_–N was determined by the nano reagent colorimetric method, TP was determined by the potassium persulphate oxidation and the molybdenum antimony anti-spectrophotometric method, TOC was determined by potassium dichromate oxidation-spectrophotometry method, Cu, Zn, Cd and Pb was determined by flame atomic absorption method. The water reduction effects of each rain garden under each rainfall event were analyzed. By analyzing the water quality and water reduction data, the loads of each pollutant under each rainfall event were calculated, and the annually pollutant loads retained by the rain garden per unit area were calculated.

### Soil sampling collection and analysis method

The collection times of soil samples were November 2018 (1st sampling), November 2019 (2nd sampling), and November 2020 (3rd sampling), that is, after the annual flood season. Soil sampling points were set at the inflow and lowest points of each rain garden. The collection of soil samples was divided into the following four steps: (i) Determined the location of the sampling point, and excavate the surface debris and 0–5 cm soil; (ii) collected soil at 5–10 cm (1000 g at each sampling point); (iii) mixed and separated the soil samples by the multiple quartering methods (500 g), put the samples in a sealed bag and put them in a low-temperature storage box. Samples were shipped to the laboratory as soon as possible and then stored at – 20 °C for preprocessing and further analysis. By analyzing soil samples, the accumulation of pollutants and the evolution of microbial communities in rain gardens under different drainage types were revealed. The physical and chemical properties of soil include soil moisture content (SMC), TN, TP, NH_3_–N, NO_3_–N, TOC, Cu, Zn, Cd, and Pb. The analyses of soil microbial communities were determined using high-throughput sequencing technology. The microbial community assay region was 16s rDNA V3 + V4, and the primer sequence was 341F/806R (ACTCCTACGGGAGGCAGCAG/GGACTACHVGGGTWTCTAAT). Microbial community analysis indicators included microbial diversity (Chao1 index, Shannon index) and microbial community structure^28^. Differences in all data were tested by analysis of variance (ANOVA). Redundancy analysis (RDA) and Procrustes analysis was used to reveal the relationship between dominant microorganisms and soil properties.

## Results and discussion

### Rainfall events monitoring of rain gardens

In the monitoring of rainfall events, not all rainfall events were monitored due to the limitation of climatic conditions and weather uncertainty. From the construction times of each rain garden to October 2020, the number of monitored rainfall events and the effect of water reduction are shown in Table 2. The precipitation of rainfall ranged from 2 to 98.15 mm across all monitored rainfall events. Since only the drainage-type rain gardens have outflows, for the water reduction effects, only RG-1A and RG-2A were compared. The results show that there are significant differences between the two rain gardens. The average water volume reduction rates of RG-1A reached more than 95%. However, RG-2A was built earlier and operated for a longer time. The runoff pollutants accumulated in RG-2A, and were internal blockage, which reduced its water volume reduction rates^29^.

**Table 2:** Rainfall events monitoring and runoff treatment effects of rain gardens.

Rain gardens	Rainfall events monitoring	Number of rainfall events	Number and rating of rainfall events*	Water reduction rate: min–max (mean)							
RG-1A/RG-1B	2015/5–2020/10	46	17 light rains, 19 moderate rains, 9 heavy rains, and 1 intense rain	36.2–100% (96.9%)							
				–							
RG-2A/RG-2B	2013/5–2020/10	49	19 light rains, 17 moderate rains, and 13 heavy rains	11.9–100% (61.1%)							
				–							

Rain gardens	Pollutant load, kg/(hm^2^·year)	Water quality indicators									
		SS	TN	TP	NH_3_–N	NO_3_–N	COD	Cu	Zn	Cd	Pb
RG-1A	L_in_	304.82	13.28	1.89	3.07	4.31	352.46	–	–	–	–
	L_out_	108.17	4.18	0.67	1.12	1.48	133.89	–	–	–	–
	L_retention_	196.66	9.11	1.22	1.95	2.82	218.57	–	–	–	–
RG-2A	L_in_	1270.80	63.07	4.67	23.36	28.03	1128.30	0.34	1.36	0.95	–
	L_out_	487.37	29.66	2.39	8.77	15.28	479.06	0.14	0.48	0.28	–
	L_retention_	783.43	33.41	2.29	14.59	12.75	649.24	0.20	0.88	0.67	–

*Rainfall ratings are classified by the National Meteorological Department according to the precipitation within 24 h, and are divided into light rain (0.1–10 mm), moderate rain (10–25 mm), heavy rain (25 – 50 mm), intense rain (> 50 mm).

Due to the differences in the area and the underlying surface of the rain gardens, the characteristics of rainfall runoff received by the rain gardens were also different. The most intuitive manifestation is that the rain gardens had different rainfall runoff inflow loads, such as SS, COD, nitrogen, and phosphorus. The inflow loads of RG-1A were significantly lower than that of RG-2A. On the one hand, because RG-1A accepted roof runoff, while RG-2A accepted road and roof runoff, the concentrations of pollutants in road runoff were much higher than that of roof^30^. On the other hand, since the area occupied by RG-1A was built relatively recently and there were fewer human activities, the impact of human activities on the pollutant load of rainfall runoff was positively correlated. The load reduction rates of RG-1A to SS, carbon (COD), nitrogen (TN, NH_3_-N, NO_3_-N), and phosphorus (TP) were basically in the range of 60 to 70%. The load reduction rates of SS, carbon, nitrogen, and phosphorus in RG-2A were unstable. This is because RG-2A has been built for a long time, and the facility has entered "old age", while the purification ability of pollutants was weak, and the effective pollutant removal ability was exerted^31^. During the rainfall monitoring processes, the concentrations of Cu, Zn, and Cd in the runoff received by RG-1A were not detected, and the concentrations of Pb were also not detected, so the analysis of the reduction effects of these loads was not carried out. According to the data that can be monitored, the reduction effects of heavy metal load of RG-2A were poor, and the load reduction rates of Cu, Zn, and Cd were about 60% ~ 70%, which were consistent with the load reduction effects of SS, COD, nitrogen, and phosphorus. In general, the purification effects of RG-1A on runoff pollutants were better than that of RG-2A.

Urban hydrological characteristics, underlying surfaces, and human activities all affected the composition and concentration of pollutants in runoff. At the same time, due to the difference in the design parameters of the rain gardens, the removal effects of pollutants were different, which ultimately determined the different loads of pollutants (SS, COD, nitrogen, phosphorus, and heavy metals) retained by the rain gardens. The design goal of the drained rain gardens was to purify and reuse stormwater^32^. The inflowing pollutants were not completely retained in the rain gardens, and some pollutants were discharged with the drainage system. In the infiltrated rain gardens, the rainfall runoff all infiltrated through the soil, which meant that all the pollutants in the inflow remained in the rain gardens. Regardless of the drainage type of rain gardens, runoff pollutants entering the rain gardens inevitably accumulated^33^. Therefore, to maintain the long-term and operation efficiency of rain gardens, it is necessary to evaluate the accumulation effects and differences of pollutants in rain gardens with different drainage types.

### Temporal changes in soil physicochemical properties

#### Soil moisture contents (SMCs)

**Figure 2:**
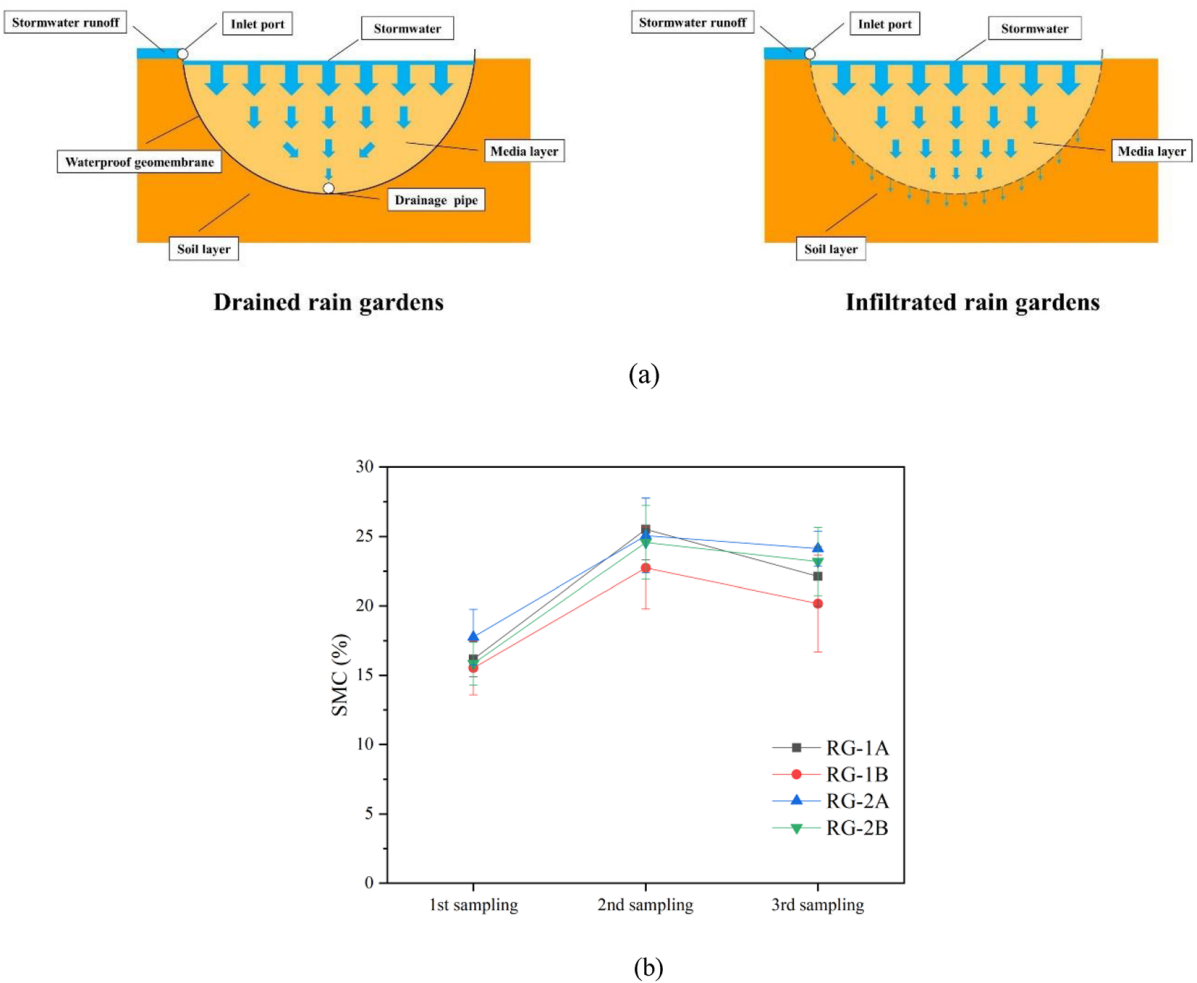
(**a**) The infiltration processes in rain gardens with different drainage types. (**b**) The SMC changes in rain gardens with different drainage types.

Due to different designs of drainage types, the infiltration degree and downward direction of runoff after entering rain gardens. Figure 2a shows the infiltration process of stormwater runoff after entering the drained and infiltrated rain gardens. In infiltrated rain gardens, stormwater runoff flows down mainly through soil, and the whole infiltration process is vertically downward. In drained rain gardens, the water flows to the drainage pipe at the bottom of the rain garden. The difference in drainage types affected the water transport in the rain garden. The changes in SMC in rain gardens with different drainage types are shown in Fig. 2b. The drained and infiltrated rain gardens showed significant differences in SMC. In three consecutive years of monitoring, the SMCs of the drained rain gardens (RG-1A and RG-2A) were always higher than that of the infiltrated rain gardens (RG-1B and RG-2B), with the SMCs of the drained ones were 102–112% of that of the infiltrated ones. Due to the different infiltration ways in rain gardens with different drainage types, runoff in infiltrated rain gardens penetrated into the deep soil, but in drained rain gardens, runoff didn’t penetrate into the soil and stayed for a longer time due to the limited outflow from drainage pipes. Stormwater runoff had longer hydraulic retention times (HRT) in drained rain gardens^18^ and gradually infiltrated down the drains in the gardens for eventual discharge. In infiltrated rain gardens, runoff gradually infiltrated downwards to achieve the goal of recharging groundwater. Since water transport in rain gardens was highly correlated with pollutant transport and transformation processes^34^, the differences in drainage types led to differences in the accumulation levels of pollutants in rain gardens.

#### Soil carbon, nitrogen, and phosphorus contents

**Figure 3:**
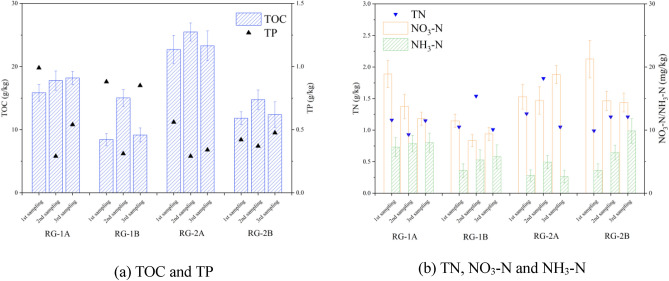
Carbon, nitrogen, and phosphorus contents in rain gardens with different drained types.

Rain gardens can efficiently receive stormwater runoff and effectively remove runoff pollutants through the soil/media-plant-microbial systems^35^. In the long-term operation of the rain gardens, the accumulation of runoff pollutants affected the contents of carbon (TOC), nitrogen (TN, NH_3_-N, NO_3_-N), and phosphorus (TP) in the soil^36^. The changes in soil carbon, nitrogen, and phosphorus in the long-term operation of rain gardens with different drainage types are shown in Fig. 3. The effects of drainage type on the soil TOC contents in the rain gardens were particularly significant, and the TOC contents of the drained rain gardens were significantly higher than that of the infiltrated rain gardens. Especially RG-2A and RG-2B, the TOC contents of RG-2A were > 1.72 times more than RG-2B, while the highest was 1.98 times (3rd sampling). The runoff intercepted in the drained rain gardens migrated to the drainage pipeline, but this also caused runoff pollutants to migrate downward, and more organic pollutants accumulated on the soil surface^37^. However, due to the long interval between the two samplings, the stormwater in the infiltrated rain gardens had enough time to slowly infiltrate, the organic carbon in the facility gradually migrated downward, and the TOC contents level of the surface layer 0–20 cm was low. The load reduction of TP in the runoff by rain gardens was mainly attributable to the reduction of runoff volume^38^. Since the purification effects of TP in runoff by the rain gardens were not very good, the changes in TP contents in soil fluctuate greatly. There was no significant correlation between drainage types and soil TP contents.

Figure 3b shows the changing trends of soil nitrogen in the rain gardens. The TN contents in the four rain gardens fluctuated greatly, but the drainage type did not have significant effects on the TN contents. TN in rain gardens was mainly affected by the runoff load caused by human activities, and the type of underlying surface^39^. However, there was no significant difference in inflow load between the rain gardens involved in this study. Therefore, the effects of different drainage types on the TN contents in rain gardens were not very large. The NH_3_-N contents in the four rain gardens had little difference. RG-1A, RG-1B, and RG-2B showed continuous accumulation trends, while the NH_3_–N contents of RG-1A did not change much, and generally showed an increase first and then decreasing trend. The NH_3_-N contents showed less volatility indicating that there was less effective nitrification in rain gardens^11^. The soil NO_3_-N contents of drained rain gardens were higher than infiltrated rain gardens. The NO_3_-N contents of RG-1A were 1.13 times (average) than that of RG-1B, and the NO_3_-N contents of RG-2A were 1.31 times (average) of RG-2B. NO_3_–N in soil was mainly removed by denitrification (denitrifying bacteria), and most denitrifying bacteria are anaerobic bacteria. Since the runoff in the infiltrated rain gardens was in the process of gradual infiltration, the denitrifying bacteria in the soil can effectively degrade NO_3_-N, so the NO_3_-N contents in the infiltrated rain gardens were low.

#### Soil heavy metals contents

**Figure 4:**
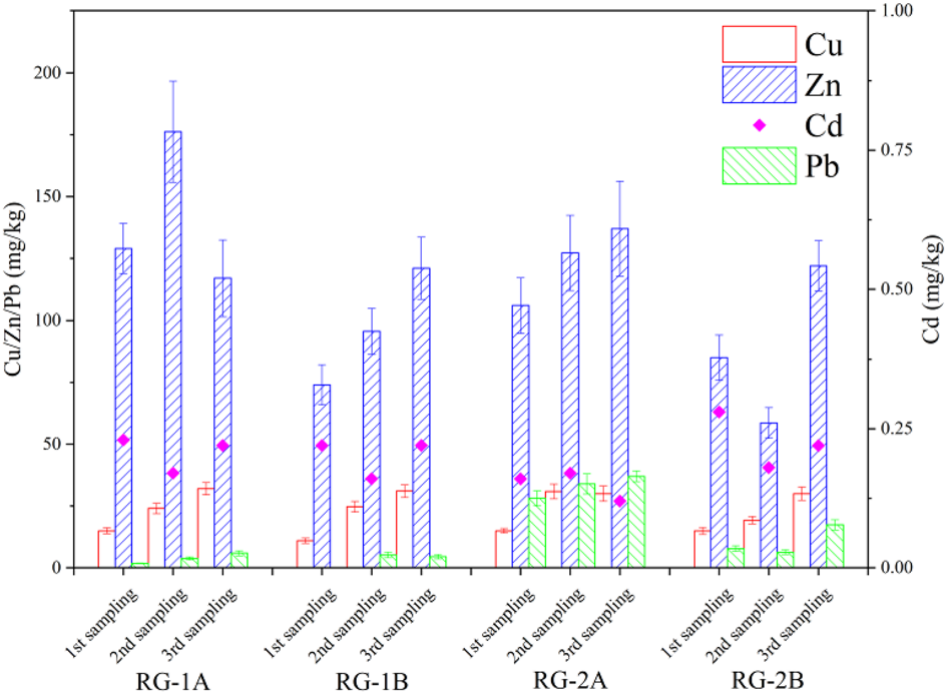
The contents of heavy metals in rain gardens with different drainage types.

Heavy metals and other pollutants contained in rainfall runoff can be quickly absorbed and removed by the soil in the rain gardens after runoff gathering in rain gardens. However, heavy metals also gradually accumulate in rain gardens^40^. Figure 4 shows the contents of heavy metals in rain gardens with different drainage types. The contents of Cu, Zn, and Pb in the four rain gardens showed gradual increase trends, and heavy metals showed accumulation characteristics under the long-term operation of the rain gardens. Comparing the effects of the drainage type on the Cu, it was found that the differences between RG-1A and RG-1B, RG-2A, and RG-2B were small, and the drainage type had no significant effects on the accumulation of Cu. The changes in Zn in rain gardens were similar to TOC, that is, the contents of Zn in drained rain gardens were significantly higher than that in infiltrated rain gardens. The contents of Cd in the four rain gardens did not change much (in the range of 0.12–0.28 mg/kg), and there was no significant accumulation. The Pb in stormwater runoff mainly comes from pollution sources such as automobile exhaust^21^, and RG-2A and RG-2B received stormwater mainly as road runoff. For this reason, there was an accumulation of Pb in RG-2A and RG-2B, and the contents of Pb gradually increased with operation time. Soil organic matter can effectively adsorb heavy metals in runoff. Since heavy metals in runoff had longer HRTs to combine with soil organic matter in drained rain gardens, the accumulation levels of heavy metals were higher than those infiltrated ones. Heavy metals are difficult to degrade in the environment, and are easily enriched in living organisms and difficulties in treatment. Therefore, the increasing heavy metal contents in rain gardens had strong harmful effects on the environment and ecosystems, and even harmed plants, microorganisms, and humans. In general, under the long-term operation of rain gardens, heavy metals showed trends of accumulation over time. The differences in drainage type led to the accumulation of Zn and Pb in drained rain gardens higher than those in infiltrated rain gardens.

### Microbial community evolution

#### Microbial diversity

**Figure 5:**
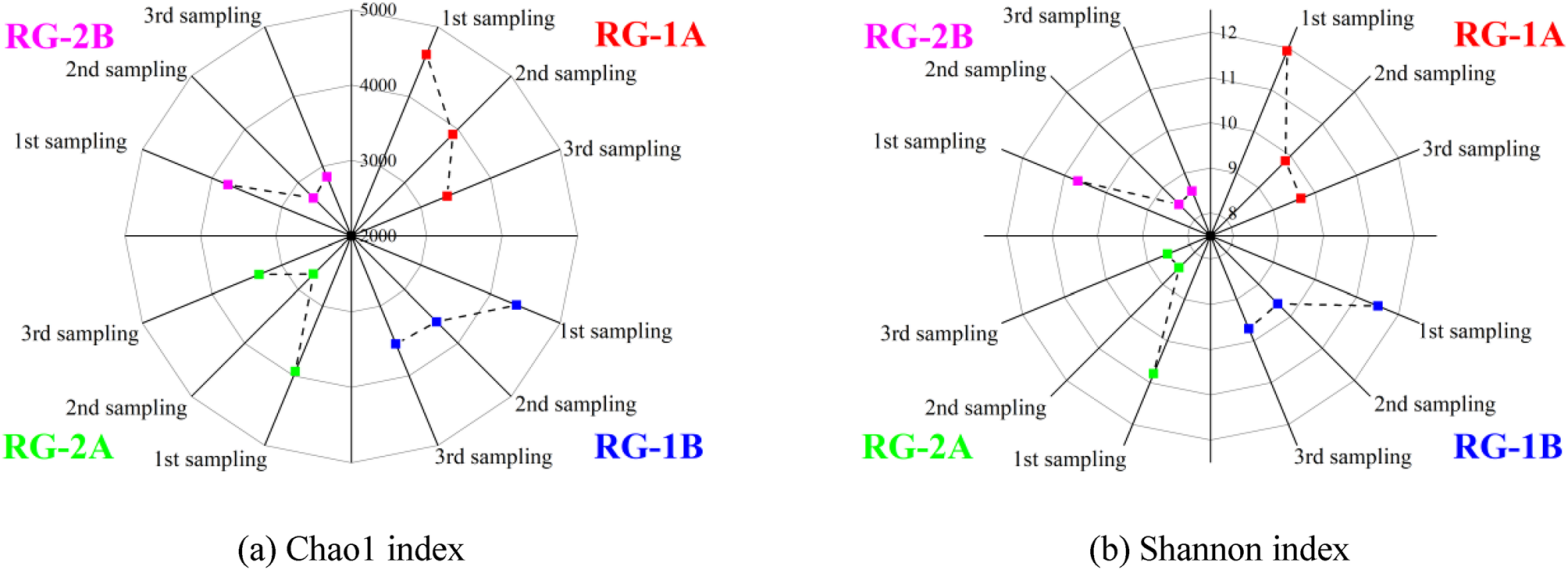
The changes in microbial diversity of rain gardens with different drainage types.

The fluctuation of the soil ecosystem affected the growth environment of soil microorganisms. If the plants and microorganisms are not in the most suitable growth environment, their degradation effect on pollutants will become worse over time, affecting the operation efficiency of rain gardens. In this study, different drainage types affected the water and pollutants migration and transformation processes in rain gardens, which were mainly reflected in the differences in SMCs and pollutant contents. The accumulation of pollutants was bound to affect the living environment of microorganisms in rain gardens and led to changes in the microbial community. Figure 5 reveals the changes in microbial diversity of rain gardens with different drainage types. With the increase in operating time, the Chao1 and Shannon indexes in the four rain gardens showed decreasing trends, and the microbial diversities in RG-2A/RG-2B were always lower than that in RG-1A/RG-1B. In the long-term operation of green infrastructure such as rain gardens, the microbial species tended to be single, while the microbial diversities were negatively correlated with the operation time^40^. Although the microbial diversities gradually decreased throughout the monitoring period, the rain gardens RG-1A and RG-1B with different drainage types also showed differences. At the first sampling (2018/11), both Chao1 and Shannon indexes in RG-1A were higher than those in RG-1B. However, by the third sampling (2020/11), the accumulation of pollutants in RG-1B, especially the accumulation of heavy metals, was lower than that of RG-1A. This resulted in significantly greater decreases in Chao1 and Shannon indexes in RG-1A than in RG-1B. Comparing RG-2A and RG-2B, the Chao1 and Shannon indexes in the two rain gardens did not show significant differences. The reason is that since the two rain gardens were built in 2012 and have been in operation for a long time, pollutants have accumulated in the rain gardens and caused a certain degree of hardening/clogging of the soil^41^. After the runoff entered the rain gardens, it was stored in the ponding layer, and the effects of the drainage types on the microbial diversities were lower than the effect of the operation time. The microbial ecosystems in the two rain gardens were completely stabilized, and the diversities were no longer reduced. Soil microbial communities (diversity and community structure) are important indexes to evaluate soil health, which can effectively reflect soil environmental changes. Due to the changes in soil properties, the soil ecosystem changes, especially the soil microorganisms are affected by the changes in soil properties. After the rain gardens were built, the differences in drainage types had significant impacts on the microbial diversities. Since the accumulation of pollutants in the drained rain gardens was significantly higher than that of the infiltrated rain gardens, it led to significantly higher reductions in microbial diversities of drained rain gardens than infiltrated rain gardens. Microbial diversities effectively reflected the pollutant accumulation level in rain gardens, and microbial diversity indexes were negatively correlated with pollutant accumulation levels. However, after the long-term operation, the most critical factor affecting the microbial diversities was the operation time of the rain gardens, and the influence of the drainage types was negligible.

#### Microbial community structure

**Figure 6:**
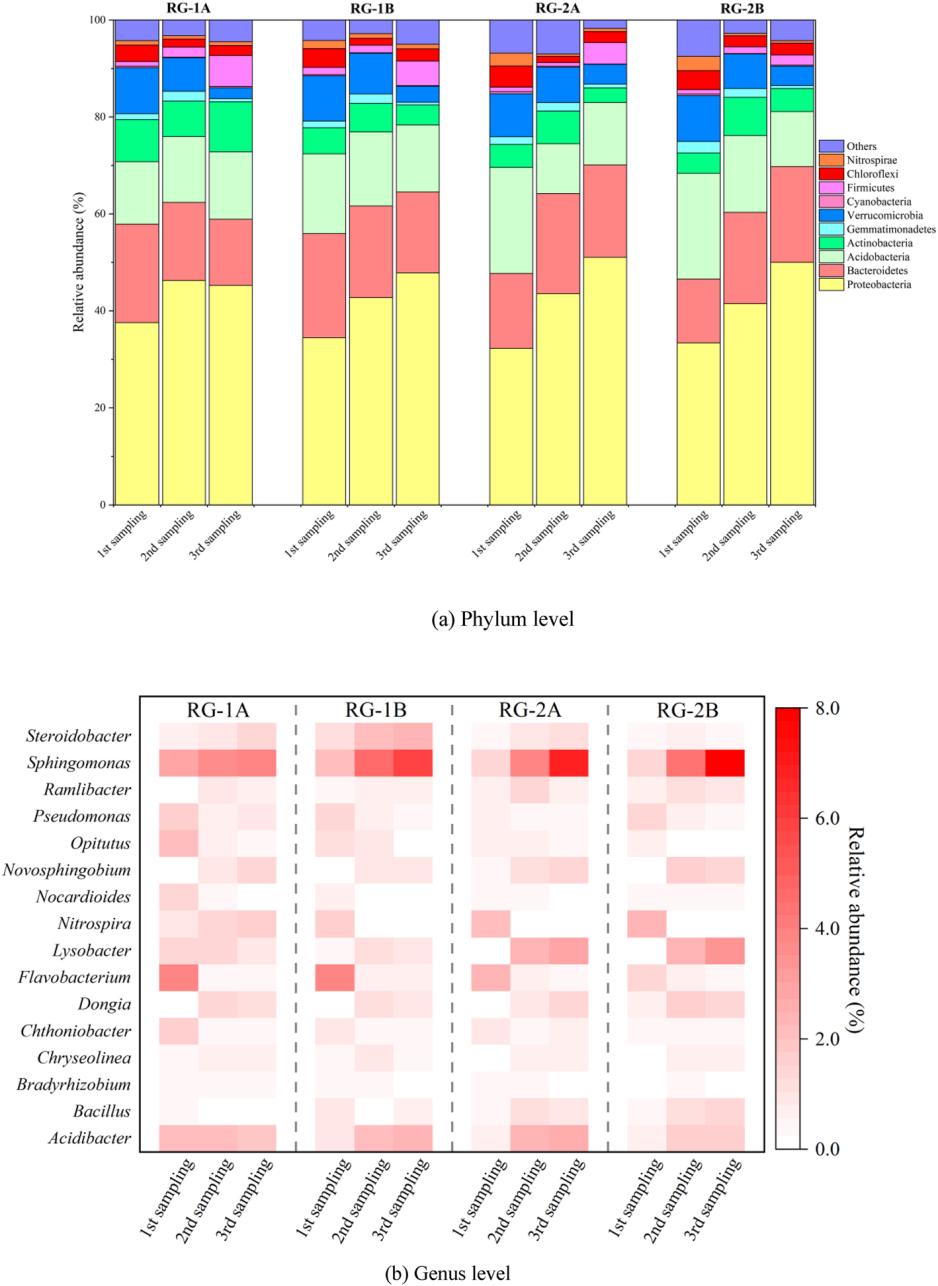
Microbial community structures of rain gardens with different drainage types.

Microbial community structures at the phylum level and genus level of rain gardens with different drainage types were analyzed, and the results were shown in Fig. 6a,b. In the four rain gardens, the relative abundances of the 10 dominant bacteria phyla accounted for more than 92% of the total sequences. The total relative abundances of *Proteobacteria* (32.27–1.06%), *Bacteroidetes* (13.18–22.30%), *Acidobacteria* (10.26–21.83%), *Actinobacteria* (3.01–10.32%) and *Verrucomicrobia* (2.25–9.77%) were more than 80%. With the increase in operation time, the relative abundances of *Proteobacteria* in the four gardens showed a trend of increasing continuously, with the least increasing degree of 7.69% (RG-1A) and the largest increasing degree of RG-2A (18.79%). *Bacteroidetes* in RG-1A and RG-1B showed decreasing trends, while RG-2A and RG-2B were on the contrary. The relative abundances of *Bacteroidetes* in the third sampling were significantly increased compared with that in the first sampling. The relative abundances of *Gemmatimonadetes* and *Verrucomicrobia* decreased while that of Firmicutes increased in the four rain gardens. For the whole monitoring period, the distribution of microbial species in RG-1A and RG-1B, RG-2A, and RG-2B did not show significant differences, and the changes of dominant bacteria at the phylum level were the same, and the influences of drainage types on microorganisms were not significant.

*Acidibacter*, *Flavobacterium*, *Nocardioides*, *Pseudomonas*, and *Sphingomonas* were dominant genera in the four rain gardens during the first sampling. However, the relative abundances of these five microorganisms showed different trends during facility operation. *Acidibacter* and *Sphingomonas* showed increasing trends in the four rain gardens, with the relative abundances of *Acidibacter* and *Sphingomonas* in the third sampling being 2.52 ~ 3.34 times and 1.40 ~ 5.38 times higher than that at the first sampling, respectively. *Sphingomonas* can metabolically grow and survive in low nutrient conditions by using organic compounds^22^, and the increasing relative abundances of *Sphingomonas* also reflect the accumulation of runoff pollutants in rain gardens^42^. Most of the accumulated pollutants were heavy metals and other harmful pollutants. The accumulation of pollutants destroyed the original habitat of the garden and made the microorganisms in the rain garden under low nutrient conditions^43^. The abundance of *Flavobacterium*, *Nocardioides*, and *Pseudomonas* decreased with the operation of the rain garden. The decrease degrees were significant for *Flavobacterium* and *Pseudomonas*, and both of these microorganisms play a key role in nitrogen and phosphorus removal^44^. The decrease in its relative abundances significantly inhibited the removal of N and P in rain gardens. The influences of different drainage types on rain gardens were mainly reflected in the difference in the infiltration mode and degree of stormwater runoff inside the facilities^28^, which mainly affected the water contents and also led to the different degrees of pollutant accumulation in the facilities.

### Analysis of dominant microorganisms and soil properties

**Figure 7:**
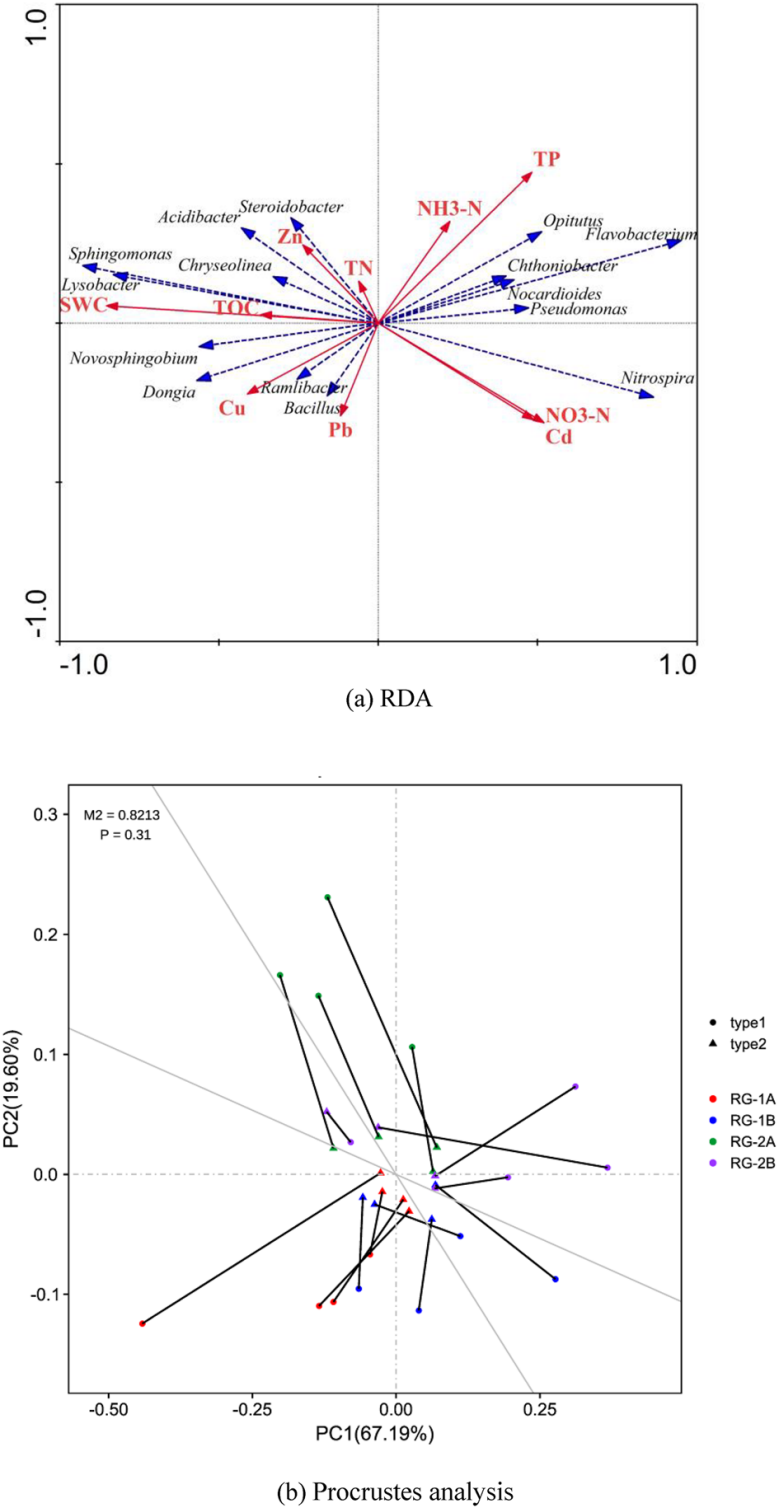
Correlation between dominant microorganisms and environmental factors in rain gardens with different drainage types.

RDA and Procrustes analysis was used to reveal the correlation between environmental factors and dominant microorganisms in rain gardens with different drainage types, and the results are shown in Fig. 7a and b. The effects of SMC and TOC on dominant microorganisms were the same, with significant positive correlations with *Lysobacter* and *Sphingomonas*, and significant negative correlations with Pseudomonas. TP was significantly negatively correlated with *Ramlibacter*, NH_3_-N was negatively correlated with *Bacillus*, NO_3_-N was negatively correlated with *Chryseolinea*, and TN was positively correlated with *Steroidobacter*. Caused of the different drainage types, the rain gardens showed the differences between SWC and TOC. The SMC and TOC of drained rain gardens were higher than those of infiltrated rain gardens in the same period. *Lysobacter* (mainly grown in soil, decaying organic matter, and freshwater, influenced by soil type and seasonal factors) and *Sphingomonas* (typical industrial pollutant and environmental pollutant degradation functional bacteria) were significantly positively correlated with SMC and TOC, which also indicated that these two functional microorganisms in the drained rain gardens were higher than that of infiltrated rain gardens. *Pseudomonas*, as one of the typical bacterial strains capable of degrading organic pollutants in the environment, has higher relative abundances in the infiltrated rain gardens due to its negative correlation with SMC and TOC. It also means that the removal ability of organic matter in the infiltrated rain gardens was stronger than that in the drained rain gardens. Cu, Zn, and Cd showed correlations with the most dominant microorganisms. Zn was positively correlated with *Steroidobacter*, Cd was negatively correlated with *Acidibacter*, and Cu was positively correlated with *Ramlibacter* and negatively correlated with *Opitutus*. Procrustes analysis is used to analyze the correlation between the dominant microorganisms and environmental factors. In Fig. 7b different colors represent different rain gardens, and the circle (type1) represents the quadrat points from the environmental factor PCA mapped in the main orthogonal axis. Triangles from the quadrat points of the dominant microorganisms’ composition PCA map points in the oblique orthogonal axis. The shorter the line segment, the smaller the residual value and the higher the consistency. M^2^ is the sum of squares of residuals, and the smaller M^2^ is, the better the consistency of the two groups of data is. The potential relationships between environmental factors and dominant microorganisms in different rain gardens showed good consistencies (P < 0.05), and were significantly related. In this study, Cu, Zn, Cd and Pb content in rain gardens was investigated. The accumulation levels of heavy metals in the drained rain gardens were higher than that in the infiltrated rain gardens, and the correlations between heavy metals and the most dominant microorganisms were significant, which means that the accumulation of heavy metals led to changes in the abundances of dominant microorganisms^45^. With the continuous increase of operation time, the accumulation level of heavy metals in the rain garden continued to rise, and the microbial communities tended to the population that could tolerate heavy metals. When microbial populations become homogeneous, the internal micro-ecosystems of the drained rain gardens were in unstable states.

### Suggestions on the construction and management of rain gardens

Different drainage types affected soil properties, which meant that rain gardens needed to be built with the actual application area in mind. In urban roadside areas, it is considered to build infiltrated rain gardens to reduce the accumulation risk of heavy metals due to the high contents of heavy metals in the runoff. In urban residential areas, it is considered to construct drained rain gardens, while the higher SWC and TOC can ensure the growth of plants, and enhance the improvement of urban green space in the living environment. Although rain gardens are widely used in urban stormwater management, their management cannot be separated from public awareness and community involvement. When rain gardens run for too long, their infiltration capacity and ecosystem level will be reduced due to blockage and other phenomena, so the public and the community should supervise and give feedback the first time. When rain gardens reach the end of their useful life, their impact on the urban environment will be negative. The efficiency evaluation of rain gardens under long-term operation can effectively improve the efficiency of rain garden management. The management should try to establish a rain garden efficiency evaluation system that includes multiple indicators. For example, in this study, it can be clearly found that soil microbial activity can effectively reflect the operation effect of rain gardens. While microbial diversity was poor and the microbial community tended to be single, the accumulation levels of heavy metal were high. Therefore, in the construction and management of rain gardens, the actual monitoring results are used to establish an engineering technology model including "reasonable construction—supervision feedback—efficiency evaluation".

## Conclusions

The study of pollutant accumulation and ecosystem changes in stormwater treatment facilities under long-term operation can effectively evaluate the operation efficiency and life of the facilities. Due to different drainage types, there were significant differences in pollutant accumulation and microbial community changes between infiltrated and drained rain gardens. SWC and TOC of drained rain gardens were always higher than those of infiltrated rain gardens, which resulted in different habitats in different rain gardens. With the increase in operation time, heavy metals in rain gardens showed gradual accumulation trends. The accumulation levels of Zn and Pb in drained rain gardens were higher than those infiltrated rain gardens due to different drainage types. The microbial diversities of drained rain gardens were lower than that of infiltrated ones, but the difference in diversities in rain gardens did not increase under long-term operation. The changes in microbial communities in different rain gardens were the same, and the influences of drainage types on microorganisms were not significant. However, the accumulation of heavy metals led to significant changes in the abundance of some dominant microorganisms. In conclusion, differences in SWC and TOC in rain gardens caused by different drainage types led to differences in pollutant accumulation levels (especially heavy metals) and microbial communities, leading to unstable states of the rain garden ecosystem under long-term operation. In the context of sustainable urban development, the results can provide a theoretical basis and technical support for urban stormwater management construction. In the construction and management of rain gardens, it is recommended to establish an engineering technology model including “reasonable construction—supervision feedback—efficiency evaluation”.

## Data Availability

The datasets analyzed during the current study are available in the [NSBI] repository [https://submit.ncbi.nlm.nih.gov/subs/sra/SUB13689301].
